# Responses of community traits and soil characteristics of *Achnatherum inebrians*-type degraded grassland to grazing systems in alpine meadows on the Qinghai-Tibet Plateau

**DOI:** 10.3389/fpls.2023.1270304

**Published:** 2023-10-06

**Authors:** Yanzhu Chen, Changlin Xu, Kaikai Ma, Qingqing Hou, Xiaojun Yu

**Affiliations:** Grassland Ecosystem Key Laboratory of Ministry of Education, Sino-U.S. Research Center for Grazing Land Ecosystem Sustainability, Grassland Pratacultural College of Gansu Agricultural University, Lanzhou, Gansu, China

**Keywords:** grazing system, *Achnatherum inebrians*, plant communities, soil microbial communities, alpine meadows

## Abstract

**Introduction:**

Scientific grazing management is of great significance for the ecological health and sustainable use of alpine meadows.

**Methods:**

To explore appropriate management methods of alpine grasslands of the Qinghai-Tibet Plateau degraded by *Achnatherum inebrians* (Hance) Keng ex Tzvele presence, we studied the effects of different grazing systems on the *A. inebrians* population, grassland vegetation community traits, soil characteristics and soil microbial community structure for cold- season grazing plus supplementary feeding pasture (CSF) and four-season open public pasture (FOP) in Tianzhu County, Gansu Province.

**Results:**

Compared with FOP, the CSF site showed significantly inhibited reproduction of *A. inebrians*, especially the crown width, seed yield and number of reproductive branches per plant were as high as 50%, significantly increased the aboveground biomass of edible forage and soil water content by 57% and 43–55%, better soil nutrients, and significantly reduced soil bulk density by 10– 29%. Different grazing systems affected the composition and diversity of soil microbial communities, with a greater effect on fungi than on bacterial flora. The most abundant phyla of bacteria and fungi were Proteobacteria and Ascomycota for CSF (by 30–38% and 24–28%) and for FOP (by 67–70% and 68–73%), and the relative abundance and species of bacterial and fungal genera were greater for CSF than FOP. The α-diversity indexes of fungi were improved, and the β-diversity of fungi was significant difference between CSF and FOP. However, the grazing utilization time was prolonged in FOP, which reduced the diversity and abundance of soil bacteria and increased soil spatial heterogeneity. The use of *A. inebrians*-type degraded grassland in the cold season, and as a winter supplementary feeding and resting ground, could effectively inhibit expansion of *A. inebrians*, promote edible forage growth, enhance grassland productivity and community stability, and improve soil structure.

**Discussion:**

The results guide healthy and sustainable utilization of *A. inebrians*-type degraded grassland in the Qinghai-Tibet Plateau.

## 1 Introduction

As a unique geographical unit of the earth, the Qinghai-Tibet Plateau is an important ecological security barrier in northwest China, with a wealth of habitat types and biological species (Fan and Fang, 2022). It has a profound impact on the surrounding area, China and the world. Alpine meadows are the most widely distributed grassland type in the Qinghai-Tibet Plateau (Niu et al., 2019), and play an important role in maintaining the ecosystem balance, including for animal husbandry development, climate regulation and water conservation (Ma et al., 2022). For decades, grazing was the dominant use in alpine meadows of the Qinghai-Tibet Plateau. However, these grasslands have also experienced spread of poisonous weeds following desertification and salinization because of the dual influence of natural and human factors, including climate change and overgrazing (Wei, 2013). This has reduced species diversity and edible forage yield, restricted the development of grassland animal husbandry and seriously threatened the livestock–soil–grassland ecosystem balance and productivity (Guo et al., 2017; Chai et al., 2019). Therefore, it is very important to study the impact of grazing on poisonous weed-type degraded grassland ecosystems in Qinghai-Tibet Plateau alpine meadows.

*Achnatherum inebrians* (Hance) Keng ex Tzvele is a perennial grass in the family Gramineae that is becoming increasingly widespread in northwest China, including in Gansu, Xinjiang, Qinghai, Tibet and Inner Mongolia (Liu et al., 2021; Liang et al., 2023). Relevant studies showed that the harmful area was 3 × 10^5^ hm^2^ in Gansu, accounting for 17.08% of the poisonous weed degraded area in the province (Guo et al., 2017). The distribution area of *A. inebrians* reached 5.3 × 10^5^ hm^2^ in Xinjiang’s natural grassland, with coverage of up to 85% in some areas (Sun et al., 2014), and its distribution area reached 5.7 × 10^5^ hm^2^ in Qinghai. In recent years, the distribution area and coverage of *A. inebrians* tended to expand in different regions, with serious economic losses to animal husbandry and also leading to grassland ecological imbalance. *A. inebrians* is the host and mutualist symbiont of the *Epichloë* endophyte, with a carrier rate of up to 100%. *Epichloë* contains high levels of ergot alkaloids (i.e., ergopeptine and ergovaline), which make it poisonous (Miles et al., 1996), and accidental feeding and starvation of juvenile or foreign livestock have caused serious losses for animal production (Yang et al., 2015). Moreover, the *Epichloë* endophyte can enhance biotic and abiotic resistance (Moon et al., 2007; Zhang et al., 2012). Li et al. (1996) showed that livestock that mistakenly ingest *A. inebrians* can quickly identify and avoid further feeding on it, so that it has a greater advantage in interspecific competition within grassland communities and continues to spread in degraded grassland, causing serious harm.

Current research on *A. inebrians* mainly focuses on control (Jin et al., 2017), characteristics (Li, 2005), seed germination (Chen et al., 2021), toxic ingredients (Liu et al., 2022) and *Epichloë* endophyte resistance (Zhang et al., 2012) in the *A. inebrians* population. Research on prevention and restoration of *A. inebrians-*type degraded grassland focuses on responses of *A. inebrians* population traits, edible forage yield and grassland community diversity to fencing, mowing, manual digging and sowing excellent forage (Li et al., 1996; Mcdaniel and Sterling, 2007; Jing et al., 2014; Yang et al., 2015). However, Sun et al. (2020) showed that long-term fencing provided no ecological and economic benefits; believed that mowing and manual digging were only suitable for *A. inebrians* grasslands with small distribution area and high growth density, and sowing was a seemingly simple but complex improvement measure. Therefore, grazing is the most important and direct utilization and management method affecting grassland biodiversity and ecosystem function, and seasonal grazing is widely used for managing degraded grassland (Zhang et al., 2023). However, there is a lack of research on the response of poisonous weeds of grasslands to grazing systems in the Qinghai-Tibet Plateau (Wei, 2013), and the comprehensive effects of different grazing systems on grassland communities, microorganisms and soil require further study.

In this study, our main objective was to study how vegetation community traits and soil characteristics of *A. inebrians*-type degraded grassland under long-term different grazing systems, explore the relationship between soil microbial community structure, soil and vegetation traits in *A. inebrians*-type grassland, and provide a theoretical basis for the restoration and health of poisonous weed-degraded alpine meadow ecosystems. More specifically we hypothesized that: (i) grassland vegetation community traits with grazing systems vary, (ii) soil physicochemical characteristics and microbial community composition change with grazing systems, (iii) the vegetation–soil–microbe are interrelated, (iv) cold-season grazing plus supplementary feeding pasture can effectively regulate *A. inebrians*-type degraded grassland.

## 2 Materials and methods

### 2.1 Study area and sampling collection

A vegetation community survey and soil sampling for this study were conducted on two separate grasslands, situated in the alpine meadow eastern edge of the Qinghai-Tibet Plateau in the administrative region of Tianzhu County, Gansu Province, China (37°11′N, 102°46′E). According to the guidance of local grassland ecological experts and herders, the *A. inebrians*-type degraded grassland was selected as the research area, which was open public grassland with basically the same vegetation as 35 years ago, with *A. inebrians* as an absolute dominant species. In 1993, it was divided into two areas: (i) one, rested in summer, had always been grazed by Tibetan sheep + yak and as a supplementary feeding site in winter, and had been fenced; and (ii) the other was still used as an open public pasture, with the same livestock grazing all year round. The distance was 20 m between cold-season grazing plus supplementary feeding pasture and four-season open public pasture, so the physical environment was similar (soil substrate and topography were the same). The altitude is 2910 m, annual mean temperature is −0.1°C, with monthly mean temperature ranging from −18.3°C in January to 12.7°C in July, and average annual accumulated temperature of 1380°C. The average annual precipitation is 416 mm, mainly concentrated in July–September and mostly terrain rain. There are only cold and warm seasons in the year, with the cold season in October–May, the warm season in June–September and the plant growth period generally April–September. The two grasslands are termed cold-season grazing plus supplementary feeding pasture (CSF) and four-season open public pasture (FOP).

### 2.2 Index determination and methods

During 15–18 August 2022, nine quadrats (1 m × 1 m) with representative and uniformly distributed vegetation were selected from each pasture for the vegetation community survey and soil sample collection. On September, 50 A*. inebrians* plants were selected from each pasture to calculate their population characteristics.

Soil samples were collected using a soil drilling core (d = 3.5 cm) divided into 0–10, 10–20 and 20–30 cm, with nine replicates in each quadrat; nine drills from each layer were mixed in bags and taken back to the laboratory. Each sample was divided into two subsamples and allocated to the following measurements: (i) soil nutrients and enzyme activity (air-dried soil) and (ii) soil microbial sequencing, for which 5–8 g of soil was dispensed into sterilized centrifuge tubes, stored on dry ice and brought back to the laboratory and stored at −80°C. Soil bulk density was sampled by a ring knife (100 cm^3^ volume) and an aluminum box dried in advance was used to determine soil water content.

#### 2.2.1 Survey of aboveground vegetation

The coverage of each species and the total coverage of the quadrat were measured by the needle-punch method. Ten plants of each species in each quadrat were randomly measured for their natural height. The aboveground biomass of each species was removed by the cutting method in each quadrat, put into an envelope, brought back to the laboratory, dried at 105°C for 30 min, then oven-dried at 65°C to constant weight and weighed. A sampling quadrat was tossed randomly 50 times into the plot and the frequency of each species recorded in each quadrat (Ren, 1998).

#### 2.2.2 Reproductive capacity of *A. inebrians*

The crown size of *A. inebrians* was determined using a steel tape measure, and then a single plant was harvested and dried it at 65°C to constant weight and weighed. Another 30 clusters of *A. inebrians* were randomly selected to count the number of reproductive branches, and the ears were removed, put into an envelope; then their length was measured, they were threshed by hand and the weight of 100 grains was determined by 0.001 g electronic balance. Thirty reproductive branches were randomly selected and their spikelets were counted; 30 spikelets were randomly selected to count the florets and seeds per spikelet.

#### 2.2.3 Soil properties

The soil bulk density, soil water content, total nitrogen, total phosphorus, available nitrogen, available phosphorus and soil organic matter were determined by ring knife method, drying method, Kjeldahl nitrogen method, molybdenum antimony anti-colorimetric method, alkaline diffusion method, NaHCO_3_ leach-colorimetry method and external heating of K_2_Cr_2_O_7_; and total potassium and available potassium were determined using a flame photometer (Model 2655−00, Digital Flame Analyzer, Cole-Parmer Instrument Company, Chicago, IL, USA), respectively (Bao, 2000). The urease, alkaline phosphatase, sucrase and catalase activities in soil were determined using sodium phenolate-sodium hypochlorite colorimetry, benzene disodium phosphate colorimetry, 3,5-dinitrosalicylic acid colorimetry and potassium permanganate titration, respectively (Guan, 1986). Soil pH was measured using a soil pH meter (PB-10, Sartorius, Göttingen, Germany). Soil samples were sent to Weikemeng Technology Co., Ltd. for Illumina MiSeq sequencing.

### 2.3 Data calculation and analysis

The Richness, Shannon–Wiener, Simpson diversity and Pielou evenness indexes were used to describe the species diversity of L and S, using the following formulas (Ma, 1994).


Pi=(C'+H'+B'+F')/4

$$D=1-\sum_{i=1}^{N}Pi^{2}$$

$$H=-\sum_{i=1}^{N}PilnPi$$

$$Pi=\frac{H}{lnN}$$

where *C*′ is relative coverage, *H′* is relative height, *F*′ is relative frequency, *B*′ is relative biomass (Lindsey, 1956), *N* is the total number of species in a quadrat and *Pi* is the importance value of species in the quadrat.

The soil microbial community used the Illumina NovaSeq platform for two-ended sequencing. The amplicon sequencing data (16S rRNA and ITS) were quality controlled, denoised and spliced to generate operational taxonomic unit, taxonomic annotation, species screening, basic statistics, significant difference comparison, α-diversity analysis, β-diversity analysis and correlation analysis using the DADA2 plugin in Qiime2 software. The species annotation information was obtained by comparing with the database [16S rRNA default Greengenes Database (version 13_8), ITS default United database (version 8.2)] The Shannon and Simpson indexes were used to assess the species diversity of samples, the Chao1 index was used to reflect species richness, and the Kruskal–Wallis method was used to determine whether there was a significant difference in an α-diversity index between the groups. Principal coordinate analysis of β-diversity was based on the weighted UniFrac distance calculation, and the significance of β-diversity was determined using Permutational multivariate analysis of variance. Besides, the closer the distance between the dots of different colors, the more similar the species composition and structure, and the closer the distance between the dots of the same color, indicating that the samples are better clustered in the group. The correlations between environmental factors and the relative abundance of soil microbiota at the phylum level were analyzed using Redundancy analysis, and the significance of the ranking axis feature values used the Monte Carlo permutation test.

All statistical analyses of vegetation community and soil physicochemical indexes were performed using SPSS software (version 19.0), including Shapiro–Wilk normal distribution assessment and independent sample t-test (*p*< 0.05). The measurement results are expressed as mean ± standard deviation. The data were plotted using Origin 2021.

## 3 Results

### 3.1 Characteristics of grassland plant communities in different pastures

#### 3.1.1 Vegetation community characteristics and plant diversity

The aboveground biomass, coverage and height of *A. inebrians* for CSF were 52.73%, 94.32% and 19.00% lower than those of FOP, respectively (*p*< 0.05); the aboveground biomass and height of edible forage in CSF were 56.82% and 123.35% higher than those of FOP, respectively (*p*< 0.05), and there was no significant difference in the sum of the coverage of each edible forage between CSF and FOP (*p* > 0.05).

The vegetation community diversity differed between the two pastures: the Simpson and Pielou indexes for CSF were 12.86% and 26.03% higher than those of FOP, respectively (*p*< 0.05), but there were no significant differences in Richness and Shannon–Wiener indexes between CSF and FOP (*p* > 0.05) (**Table 1**).

**Table 1:** Vegetation community characteristics and plant diversity of different pastures.

Vegetation characteristics	Cold-season grazing plus supplementary feeding pasture	Four-season open public pasture
Aboveground biomass of *A. inebrians* (g)	22.09 ± 5.83b	146.73 ± 16.46a
Aboveground biomass of edible forage (g)	201.25 ± 29.63a	128.33 ± 26.91b
Coverage of *A. inebrians* (%)	3.30 ± 1.53b	58.60 ± 7.09a
Sum of sub-coverage of edible forage (%)	113.00 ± 7.21a	100.60 ± 6.43a
Average height of *A. inebrians* (cm)	53.50 ± 3.50b	66.00 ± 0.96a
Average height of edible forage (cm)	19.61 ± 3.22a	8.78 ± 1.73b
Richness index	7.67 ± 0.58a	9.00 ± 1.00a
Simpson index	0.79 ± 0.04a	0.70 ± 0.02b
Shannon–Wiener index	1.87 ± 0.26a	1.60 ± 0.13a
Pielou index	0.92 ± 0.10a	0.73 ± 0.07b

The data are expressed as mean ± standard deviation, and different lowercase letters after the standard deviation number indicate a significant difference between the two pastures (p< 0.05).

#### 3.1.2 Reproductive ability of *A. inebrians*

The length of the long and minor axes, dry weight and crown width of *A. inebrians* were 36.28%, 35.76%, 36.13% and 59.26% lower for CSF compared to FOP, respectively. The reproduction of *A. inebrians* was significantly lower for CSF than FOP, especially the seed yield and number of reproductive branches per plant were as high as 52% (*p*< 0.05) (**Table 2**).

**Table 2:** Reproductive ability of *A. inebrians* in different pastures.

Indexes	Cold-season grazing plus supplementary feeding pasture	Four-season open public pasture
Length of the long axes	30.44 ± 1.54b	47.48 ± 0.89a
Length of the minor axes	23.89 ± 0.73b	37.19 ± 0.51a
Dry weight	12.90 ± 1.21b	20.19 ± 3.57a
Crown width	754.67 ± 82.23b	1852.33 ± 36.81a
Ears length (cm)	11.29 ± 0.55b	13.08 ± 0.47a
Seed yield (kg·hm^−2^)	35.27 ± 1.63b	74.11 ± 2.67a
Number of reproductive branches per plant	5.97 ± 0.35b	12.60 ± 0.26a
Spikelets per reproductive branch	32.50 ± 1.15b	38.63 ± 1.47a
Number of florets per spikelet	6.62 ± 0.06b	9.18 ± 0.09a
Number of seeds per spikelet	13.17 ± 0.45b	18.63 ± 0.57a
1000-grain weight (g)	0.98 ± 0.05b	1.13 ± 0.01a

The data are expressed as mean ± standard deviation, and different lowercase letters after the standard deviation number indicate a significant difference between the two pastures (p< 0.05).

### 3.2 Soil properties in different pastures

#### 3.2.1 Soil physicochemical properties

**Figure 1:**
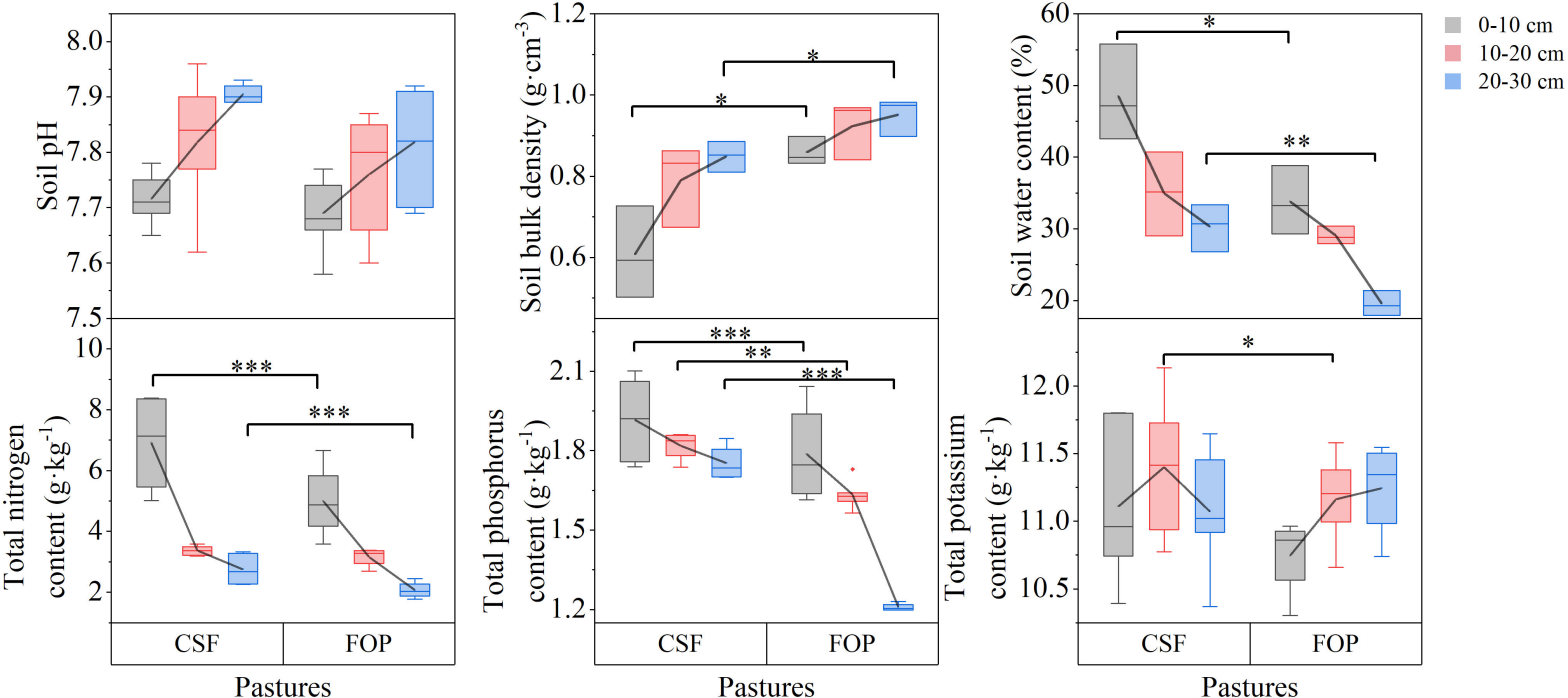
Soil physicochemical characteristics and total nutrients of different pastures. CSF and FOP are cold-season grazing plus supplementary feeding pasture and four-season open public pasture, respectively. *, ** and *** Significant at the 0.05, 0.01 and 0.001 probability level.

**Figure 2:**
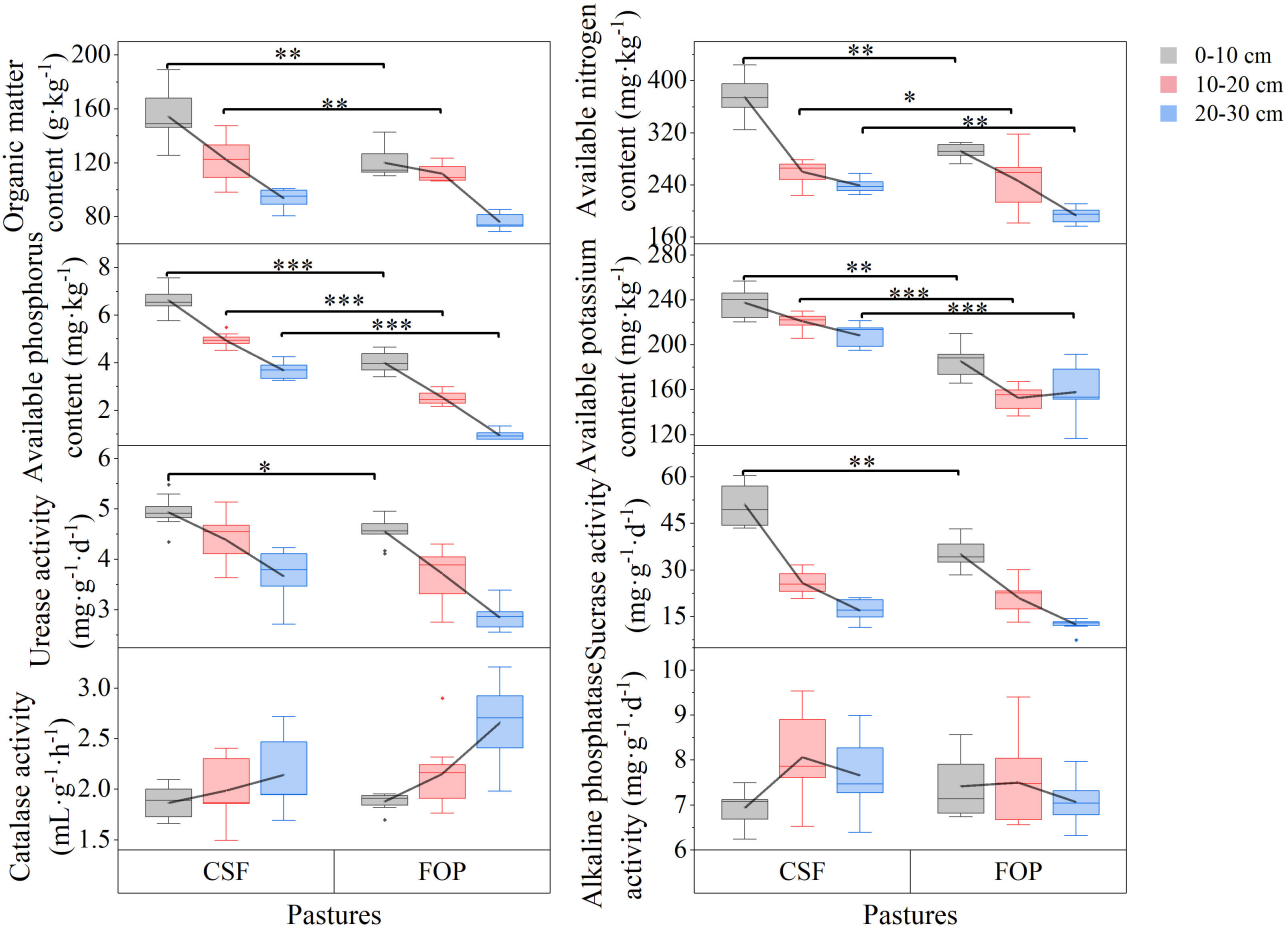
Soil available nutrients and enzyme activities of different pastures. CSF and FOP are cold-season grazing plus supplementary feeding pasture and four-season open public pasture, respectively. *, ** and *** Significant at the 0.05, 0.01 and 0.001 probability level.

Soil pH and bulk density tended to increase with greater soil depth in both pastures. However, soil water content, nutrients and organic matter tended to decrease (**Figures 1**, **2**). Compared with FOP, CSF had significantly lower bulk density (by 29.46% and 10.53%) and greater soil water content (by 43.54% and 55.10%) for 0–10 and 20–30 cm, respectively (*p*< 0.05). Besides, CSF had significantly greater total nitrogen and total phosphorus in 0–10 cm (by 39.52% and 5.02%, respectively) and in 20–30 cm (by 33.59% and 45.23%), and had significantly greater total phosphorus and total potassium by 11.16% and 2.06% in 10–20 cm, respectively (*p*< 0.05). Compared with FOP, CSF had significantly greater soil organic matter, available nitrogen, available phosphorus and available potassium in 0–10 cm (by 29.67%, 28.45%, 68.42% and 23.77%, respectively) and 10–20 cm (by 10.68%, 11.25%, 93.75% and 31.20%, respectively). There were no significant differences for pH in the three soil layers, for bulk density, soil water content, total nitrogen and total phosphorus in 10–20 cm, for soil organic matter in 20–30 cm (*p* > 0.05).

#### 3.2.2 Soil enzyme activities

The urease and sucrose activities tended to decrease with greater soil depth, which was opposite to the trend of catalase activity; and alkaline phosphatase activity tended to initially increase and then decrease in both pastures (**Figure 2**). Compared with FOP, CSF had significantly greater urease and sucrose activities by 8.31% and 45.71% only in 0–10 cm, respectively (*p*< 0.05). There were no significant differences in activities of the four enzymes in the other soil layers (*p* > 0.05).

### 3.3 Soil microbial community structure in different pastures

#### 3.3.1 Soil microbial community composition

##### 3.3.1.1 Bacterial community

**Figure 3:**
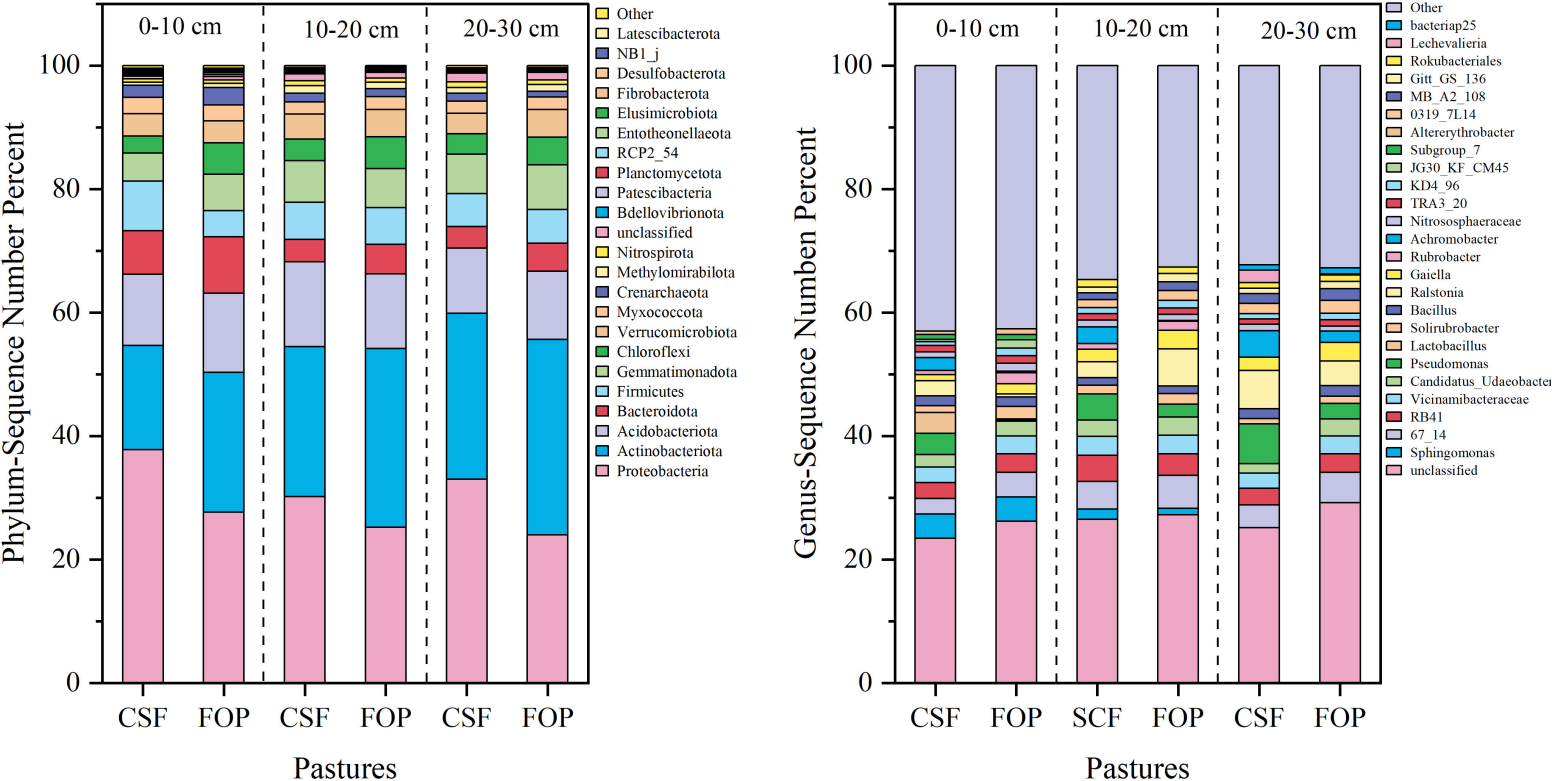
Relative species abundance of soil bacteria at phylum and genus levels in different pastures. CSF and FOP are cold-season grazing plus supplementary feeding pasture and four-season open public pasture, respectively.

The dominant bacterial phyla were Proteobacteria, Actinobacteriota and Acidobacteriota, but their relative abundances varied. The highest relative abundance was for Proteobacteria in 0–10, 10–20 and 20–30 cm, which was 37.78%, 30.16% and 32.99% in CSF, and 27.67%, 25.20% and 24.00% in FOP, respectively – all were higher for CSF than FOP. This was followed by Actinobacteriotaiota, with relative abundances of 16.87%, 24.33% and 26.91% in 0–10, 10–20 and 20–30 cm for CSF, respectively, and 22.68%, 28.96% and 31.67% for FOP. A total of 25 genera were detected in CSF and FOP, and nearly 30% of the bacterial taxa could not be classified clearly into any genus (i.e., designated unclassified). *Sphingomonas* was the dominant genera in 0–10 cm in CSF and FOP, with relative abundances of 3.91% and 3.94%, respectively, 67_14 and *Ralstonia*, *Pseudomonas* and 67_14 were the dominant genera in 10–20 and 20–30 cm, which was 4.51% and 6.04%, and 6.48% and 4.88%, respectively (**Figure 3**).

##### 3.3.1.2 Fungal community

**Figure 4:**
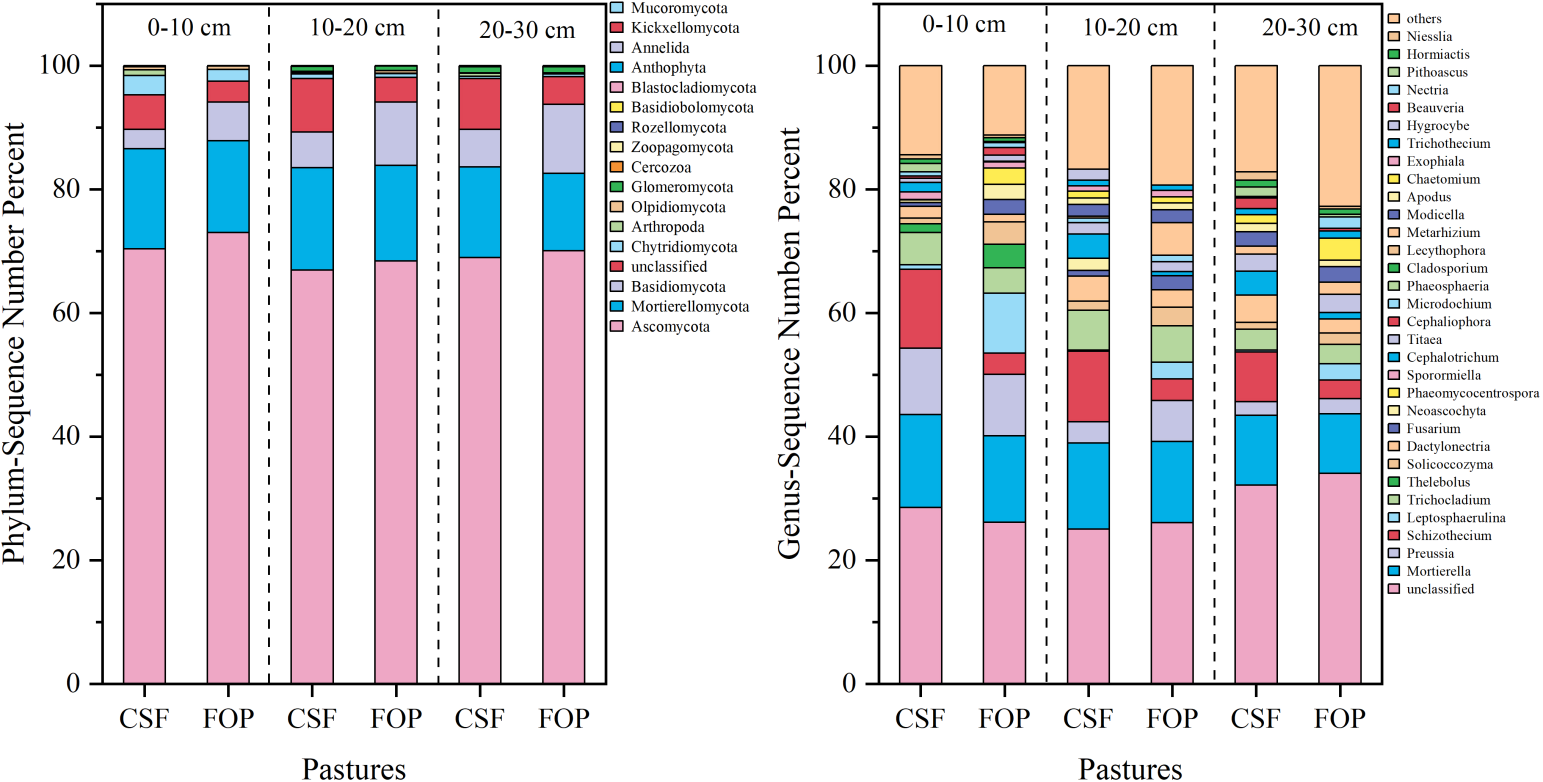
Relative species abundance of soil fungi at phylum and genus levels in different pastures. CSF and FOP are cold-season grazing plus supplementary feeding pasture and four-season open public pasture, respectively.

The dominant fungal phyla were Ascomycota, Mortierellomycota and Basidiomycota, and their relative abundances varied. The highest relative abundance was for Ascomycota in 0–10, 10–20 and 20–30 cm, with 70.38%, 66.93% and 68.95% in CSF, respectively, and 73.03%, 68.42% and 70.1% in FOP – all were lower in CSF than FOP. This was followed by Mortierellomycota, with relative abundances of 16.20%, 16.59% and 14.68% for CSF, respectively, and 14.83%, 15.42% and 12.49% for FOP. A total of 32 genera were detected in CSF and FOP, with nearly 35% of the fungal taxa not able to be classified clearly into any genus (i.e., unclassified). *Mortierella* was the dominant genus in 0–10, 10–20 and 20–30 cm for CSF with 15.00%, 11.23% and 13.11%, respectively, and 13.95%, 13.98% and 9.61% for FOP (**Figure 4**).

#### 3.3.2 Soil microbial community diversity

##### 3.3.2.1 α-diversity

**Figure 5:**
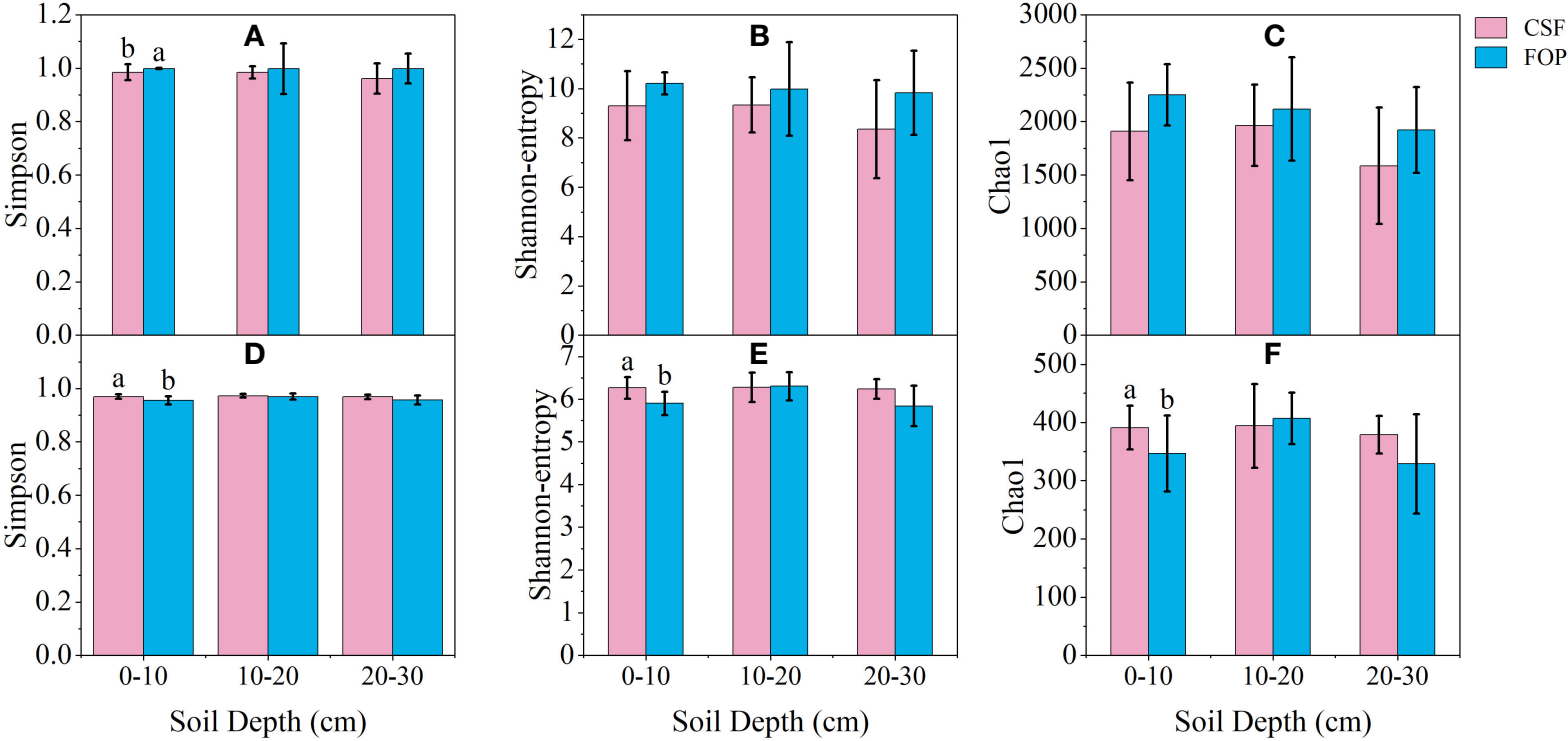
The α-diversity indexes of soil bacterial **(A-C)** and fungal **(D-F)** communities in different pastures. CSF and FOP are cold-season grazing plus supplementary feeding pasture and four-season open public pasture, respectively. Different letters indicate significant variation at the level of *p*=0.05.

Bacterial α-diversity was overwhelmingly dominant in CSF and FOP, with the Shannon and Chao1 indexes significantly higher than those for fungi (*p*< 0.05). In the bacterial community, the Simpson index for FOP was significantly higher than for CSF in 0–10 cm by 1.41% (*p*< 0.05). In the fungal community, the Shannon, Simpson and Chao1 indexes for CSF were significantly higher than for FOP in 0–10 cm (*p*< 0.05), being 6.13%, 1.5% and 12.85% higher, respectively. There was no significant differences for α-diversity indexes in the other soil layers between CSF and FOP (*p* > 0.05). In addition, the α-diversity indexes were higher for FOP compared to CSF in the three layers of soil for bacteria, and for the Simpson index in 10–20 cm for fungi. The α-diversity indexes were higher for fungi in 0–10 and 20–30 cm of CSF compared to FOP (**Figure 5**).

##### 3.3.2.2 β-diversity

**Figure 6:**
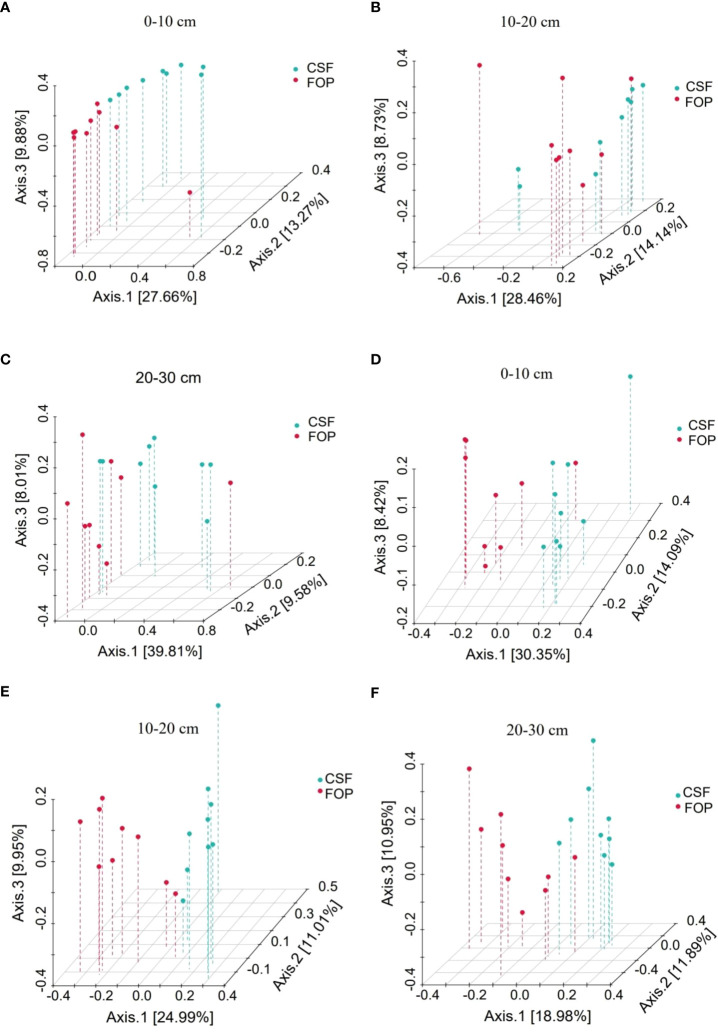
PCoA of β-diversity of soil bacterial **(A–C)** and fungal **(D–F)** communities in different pastures. CSF and FOP are cold-season grazing plus supplementary feeding pasture and four-season open public pasture, respectively. – This applies in other tables and figures below.

The Bray–Curtis Permutational multivariate analysis of variance showed a significant difference in 0–10 cm for bacteria (*p*< 0.05), but no significant difference in deeper soil between the two pastures (*p* > 0.05). The fungal communities significantly differed in the three soil layers between CSF and FOP (*p*< 0.05) (**Figure 6**; **Table 3**).

**Table 3:** Analysis of β-diversity between groups based on Permanova.

	Bacteria			Fungi		
Soil depth (cm)	0-10	10-20	20-30	0-10	10-20	20-30
Group 1	CSF	CSF	CSF	CSF	CSF	CSF
Group 2	FOP	FOP	FOP	FOP	FOP	FOP
Sample number	18	18	18	18	18	18
pseudo-F	2.093	1.6193	1.3813	5.886	3.899	2.771
*p*	0.005	0.079	0.162	0.001	0.001	0.001

CSF and FOP are cold-season grazing plus supplementary feeding pasture and four-season open public pasture, respectively.

### 3.4 Vegetation–soil–microorganism interrelationships in different pastures

**Figure 7:**
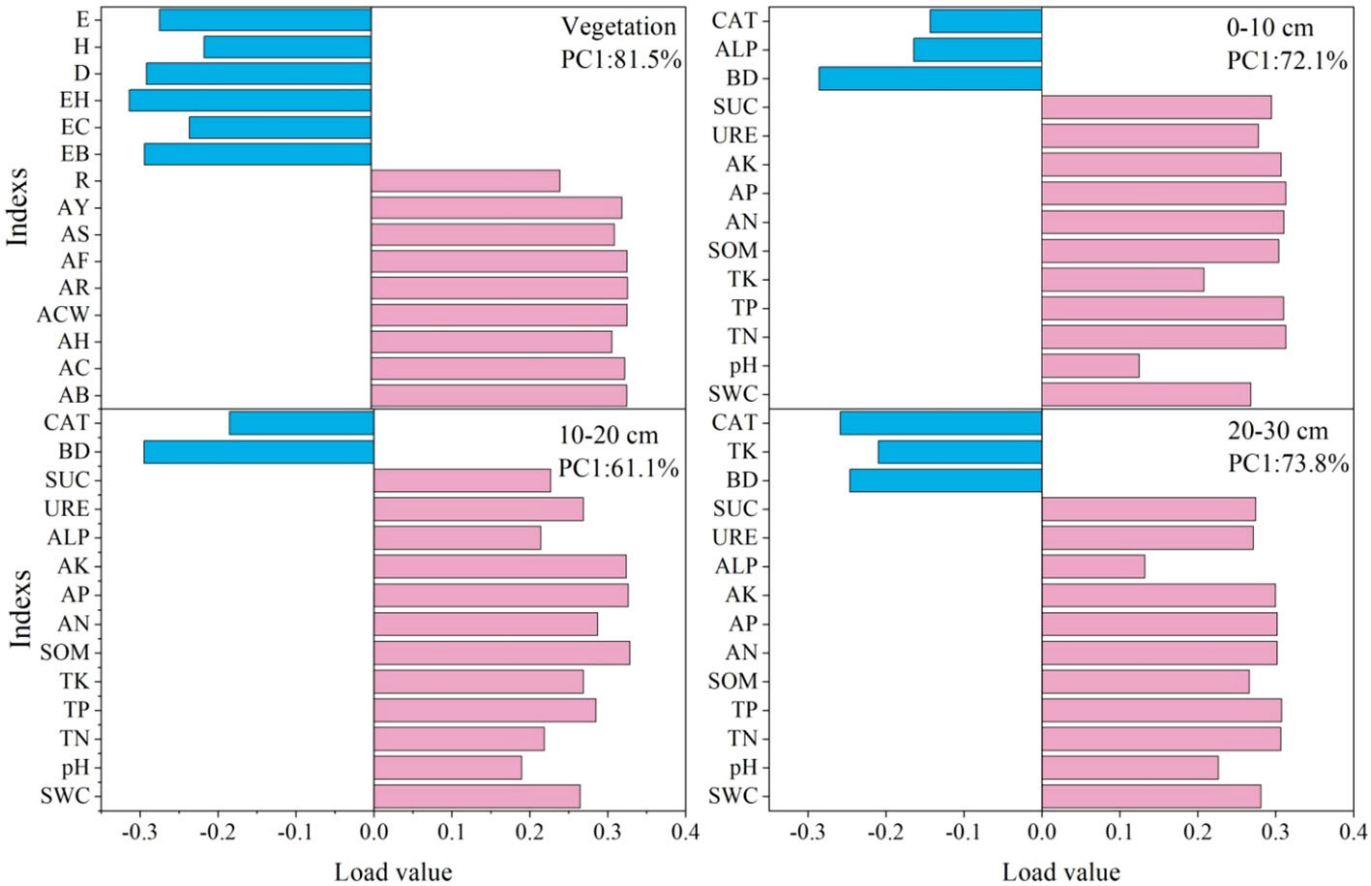
Vegetation community traits and soil characteristics of PC1 (Principal component analysis) axis load value. AB, AC, AH, ACW, AF, AR, AS, AY represent aboveground biomass, coverage, height, crown width, number of florets per spikelet, number of reproductive branches, spikelet per reproductive branches, seed yield of *A. inebrians* in turn; EB, EC, EH represent aboveground biomass, coverage and average height of edible forage in turn. E, Pielou evenness; H, Shannon–Wiener; D, Simpson diversity; BD, SWC, TN, TP, TK, SOM, AN, AP, AK, ALP, URE, SUC and CAT represent soil bulk density, water content, total nitrogen, total phosphorus, total potassium, soil organic matter, available nitrogen, available phosphorus, available potassium, alkaline phosphatase activity, urease activity, sucrase activity and catalase activity in turn. – This applies in other tables and figures below.

Vegetation communities and soil physicochemical indexes were screened using Principal component analysis (**Figure 7**). The vegetation and soil factors were screened by PC1 load values greater than 0.28 and 0.30: the important vegetation factors were aboveground biomass, coverage crown width, number of reproductive branches and seed yield of *A. inebrians*. The important soil factors were total nitrogen, total phosphorus, soil organic matter, and soil available nutrients in 0–10 cm; soil organic matter, available phosphorus and available potassium in 10–20 cm; and total nitrogen, total phosphorus and soil available nutrients in 20–30 cm.

#### 3.4.1 Correlations among soil bacteria, vegetation and soil physicochemical properties

**Figure 8:**
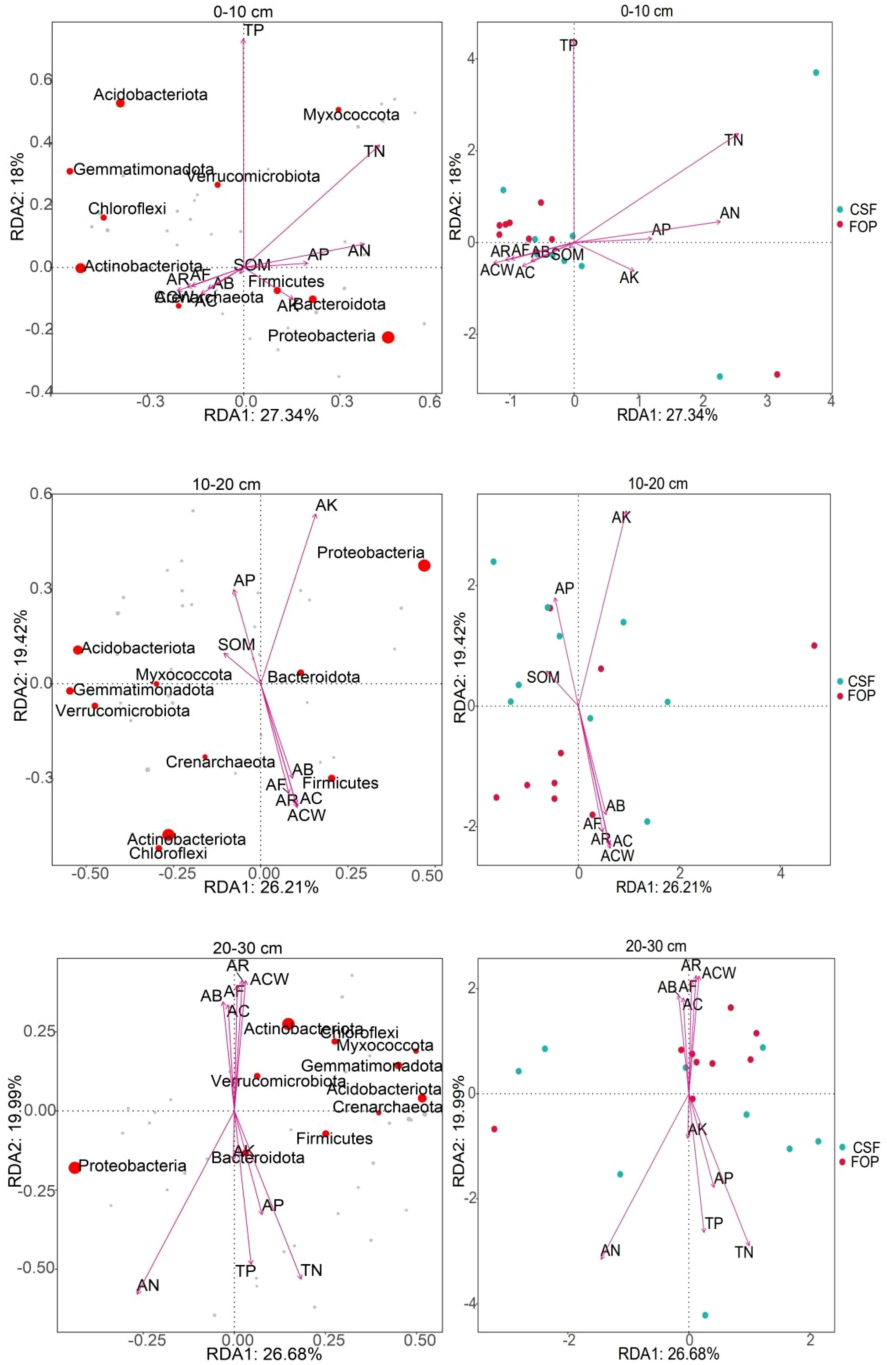
RDA of soil bacterial communities at the phylum level and environmental factors in different pastures.

The cumulative contribution rates of the first and second axes were 45.34%, 45.63% and 46.67% in 0–10, 10–20 and 20–30cm, respectively (**Figure 8**) . The most abundant phyla were Proteobacteria, Actinobacteriota, Acidobacteriota and Bacteroidota, in 0–10 cm, which had large positive correlations with *A. inebrians* reproductive capacity; and Proteobacteria and Bacteroidota had high positive correlations with soil available nutrients. Proteobacteria, Acidobacteriota and Actinobacteriota were the most abundant phyla in 10–20 cm, which were influenced by available potassium, soil organic matter and crown width of *A. inebrians*, respectively. Proteobacteria and Actinobacteriota were the most abundant in 20–30 cm, with positive correlations with available nitrogen and seed yield of *A. inebrians*. Soil bacteria were most strongly correlated with the *A. inebrians* reproductive capacity and soil organic matter in 0–10 cm of CSF and FOP. In 10–20 cm, soil bacteria were strongly correlated with soil organic matter, available phosphorus and potassium in CSF, and seed yield, number of florets per spikelet, coverage and number of reproductive branches of *A. inebrians* in FOP. Soil bacteria were also highly positively correlated with crown width, number of reproductive branches, number of florets per spikelet of *A. inebrians* in 20–30 cm of FOP, but the dispersion of CSF sample points was high. Combined with a Monte Carlo permutation test, soil bacteria were significantly correlated with available nitrogen only in 20–30 cm (*p*< 0.05), and had no significant correlations with each factor in the remaining two layers (*p* > 0.05) (**Table 4**).

**Table 4:** Monte Carlo permutation test between soil bacteria, vegetation and soil physicochemical properties.

Soil layers	Indexes	RDA1	RDA2	r^2^	*p*-value
0–10 cm	AB	−0.8555	−0.5178	0.0508	0.8111
	AC	−0.8414	−0.5404	0.0621	0.7256
	ACW	−0.9406	−0.3395	0.0864	0.5462
	AR	−0.9418	−0.3363	0.0736	0.6352
	AF	−0.9384	−0.3454	0.0679	0.6867
	TN	0.7339	0.6793	0.2267	0.1224
	TP	−0.0021	1.0000	0.2875	0.0600
	SOM	−0.5102	−0.8600	0.0092	0.9500
	AN	0.9808	0.1952	0.1508	0.2854
	AP	0.9977	0.0683	0.0787	0.6087
	AK	0.8332	−0.5530	0.0728	0.6302
10–20 cm	AB	0.2924	−0.9563	0.1670	0.2529
	AC	0.2581	−0.9661	0.2114	0.1699
	ACW	0.2571	−0.9664	0.2165	0.1534
	AR	0.2585	−0.9660	0.2105	0.1629
	AF	0.2261	−0.9741	0.1900	0.1999
	SOM	−0.7362	0.6768	0.0762	0.5557
	AP	−0.2502	0.9682	0.1647	0.2594
	AK	0.2815	0.9596	0.2991	0.0725
20–30 cm	AB	−0.0893	0.9960	0.1891	0.2114
	AC	−0.0518	0.9987	0.1833	0.2259
	ACW	0.0751	0.9972	0.2255	0.1484
	AR	0.0523	0.9986	0.2261	0.1444
	AF	0.0201	0.9998	0.2172	0.1579
	TN	0.3264	−0.9452	0.3070	0.0555
	TP	0.0949	−0.9955	0.2661	0.0905
	AN	−0.4184	−0.9083	0.3481	0.0410
	AP	0.2269	−0.9739	0.1837	0.2209
	AK	−0.0193	−0.9998	0.0853	0.5297

#### 3.4.2 Correlations among soil fungi, vegetation and soil physicochemical properties

**Figure 9:**
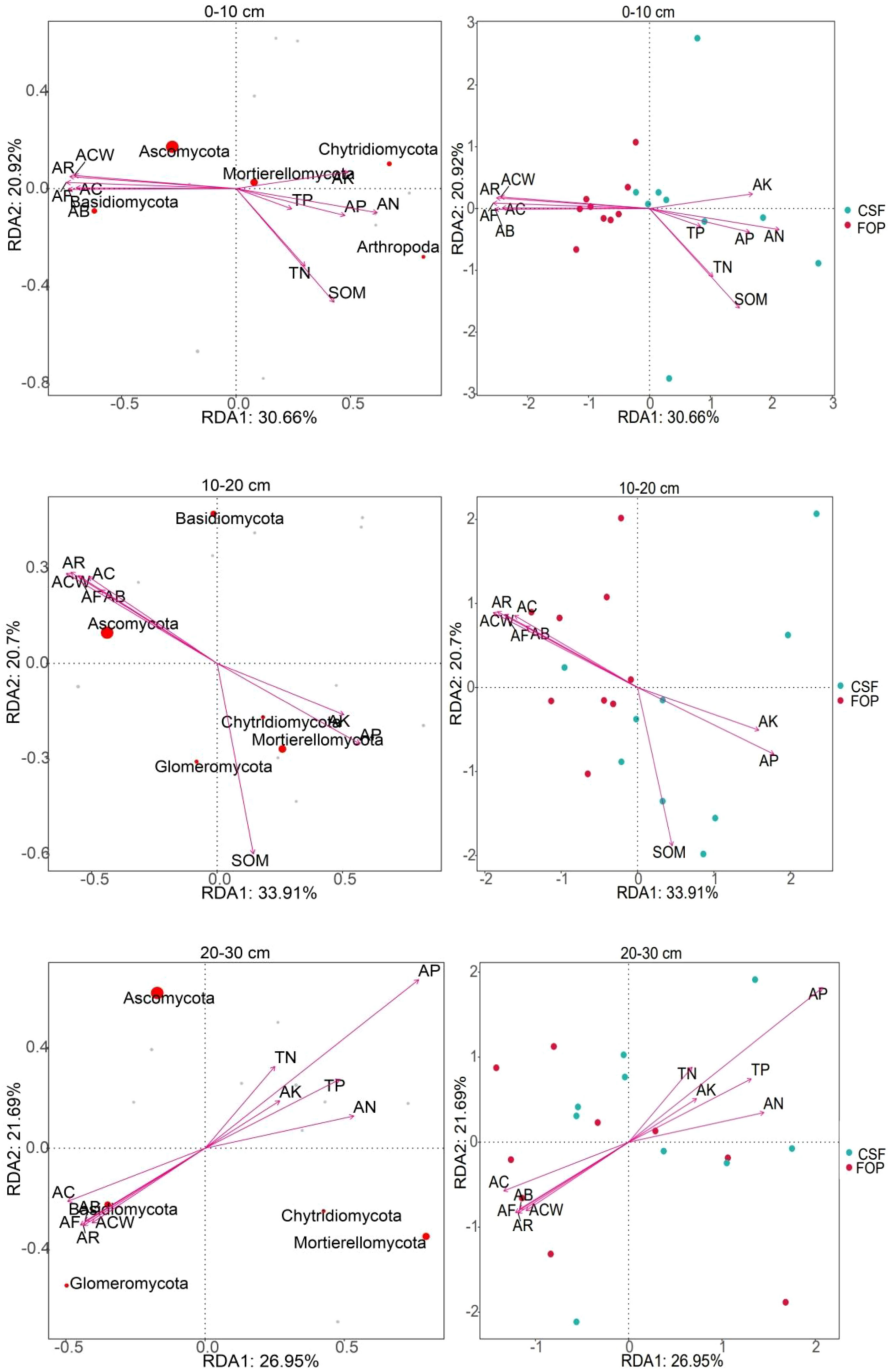
RDA of soil fungal communities at the phylum level and environmental factors in different pastures.

The cumulative contribution rates of first and second axes were 51.58%, 54.61% and 48.64% in 0–10, 10–20 and 20–30 cm, respectively (**Figure 9**). The most abundant phyla were Ascomycota and Mortierellomycota in 0–10 cm, both with high positive correlations with number of reproductive branches of *A. inebrians* and available potassium. Ascomycota and Mortierellomycota were the most abundant phyla in 10–20 cm, and Ascomycota was positively correlated with the *A. inebrians* population, and Mortierellomycota was strongly positive correlations with soil organic matter, available phosphorus and potassium. The fungal phyla with high abundance in 20–30 cm was consistent with abundances in shallower soil but were not highly correlated with each factor. Soil fungi were strongly correlated with soil chemical properties in CSF, and *A. inebrians* population traits in FOP. Sample point dispersion was high in 20–30 cm of CSF and FOP. Combined with the Monte Carlo permutation test, for 0–10 cm, fungi were significantly correlated with all factors except total phosphorus (*p*< 0.05), and very significantly correlated with aboveground biomass, coverage, crown width, number of reproductive branches and number of florets per spikelet of *A. inebrians*, soil organic matter and available nitrogen (*p*< 0.01); for 10–20 cm there were very significant correlations of fungi with all factors (*p*< 0.01). There were no significant correlations in 20–30 cm (*p* > 0.05) (**Table 5**).

**Table 5:** Monte Carlo permutation test between soil fungi, vegetation and soil physicochemical properties.

Soil layers	Indexes	RDA1	RDA2	r^2^	*p*-value
0–10 cm	AB	-1.0000	−0.0046	0.5408	0.0005
	AC	-1.0000	0.0042	0.5157	0.0005
	ACW	−0.9971	0.0766	0.5230	0.0005
	AR	−0.9977	0.0679	0.5372	0.0005
	AF	−0.9994	0.0341	0.5476	0.0005
	TN	0.6863	−0.7273	0.3236	0.0480
	TP	0.9461	−0.3238	0.1896	0.2144
	SOM	0.6757	−0.7372	0.4670	0.0065
	AN	0.9875	−0.1578	0.4606	0.0065
	AP	0.9741	−0.2261	0.3601	0.0220
	AK	0.9904	0.1385	0.3649	0.0275
10–20 cm	AB	−0.8947	0.4467	0.4449	0.0085
	AC	−0.8830	0.4694	0.4979	0.0040
	ACW	−0.9045	0.4264	0.5708	0.0005
	AR	−0.8973	0.4414	0.5580	0.0010
	AF	−0.8947	0.4467	0.5308	0.0010
	SOM	0.2340	−0.9722	0.5297	0.0055
	AP	0.9136	−0.4066	0.5339	0.0015
	AK	0.9524	−0.3047	0.4553	0.0095
20–30 cm	AB	−0.8296	−0.5584	0.1447	0.3073
	AC	−0.9193	−0.3935	0.1496	0.2914
	ACW	−0.8069	−0.5907	0.1400	0.3238
	AR	−0.8179	−0.5753	0.1488	0.2984
	AF	−0.8258	−0.5639	0.1508	0.2924
	TN	0.6103	0.7922	0.1143	0.4113
	TP	0.8700	0.4931	0.1549	0.2824
	AN	0.9724	0.2333	0.1531	0.2829
	AP	0.7530	0.6580	0.2836	0.0845
	AK	0.8179	0.5754	0.0913	0.4763

## 4 Discussion

### 4.1 Effects of grazing system on plant community characteristics

The ecological restoration of poisonous weed-type degraded grasslands is not only reflected in the poisonous weeds themselves but also in other plants and environmental changes in grassland communities. This study focused on *A. inebrians*, edible forage and community diversity, and attempted to study the vegetation community’s changes in different pastures from multiple perspectives. The aboveground biomass and height of edible forage in the CSF were significantly better than in the FOP, while *A. inebrians* performed the opposite, and had the strongest inhibition of its coverage. Wang (1962) studied the “camping circle” of sheep in Tianzhu grassland in Gansu Province and found that the grass yield and the proportion of Gramineae with excellent quality were increased significantly compared with outside the circle, and poisonous and pest grasses decreased significantly, consistent with our results. The reasons were that resting from grazing during the pasture rejuvenation period protected the plant’s underground organs from being trampled by livestock, and resting from grazing during the pasture vigorous growth period prevented livestock feeding on plants with good palatability, and so these plants had stronger community competitiveness than plants with poor palatability (Yao, 2019). Thus, resting from grazing provided more abundant grass resources for grazing in the cold season, and was conducive to the vegetation renewal of plants with bud bank as their vegetative propagation mode of potential population. Zhang et al. (2018) showed that moderate grazing affected community structure and interspecific relationships, and that the competitiveness of general dominant species was inhibited by grazing, and the coexistence of dominant species and grazing-tolerant species improved grassland productivity and stability.

*A. inebrians* is the dominant grass species in degraded alpine meadows, the study of its response to different grazing systems plays an important role in the sustainable use and conservation of species diversity in *A. inebrians*-type degraded grassland (Zhang L. et al., 2019). Our studies showed that the *A. inebrians* developed adaptive strategies for different grazing systems, and the population characteristics, seed yield and composition factors had significant differences. The selective feeding of livestock can regulate the competition between liking and not liking forage, inhibit the forage-hating and enhance the forage-loving, and then affect the community structure and function and grassland health (Hou and Yang, 2006). Continuous grazing throughout the seasons, without giving the grassland a chance to recuperate, and frequent gnawing of livestock reduces the biomass of edible forage, affects its material energy accumulation and so affects its growth, development and reproduction, and this gave *A. inebrians* an advantage in competition with excellent plant species and so its cluster diameter increased, effective photosynthetic area was larger, and more energy accumulated, which could be used for the production of more constituent factors. Studies in the Qinghai-Tibet Plateau also showed that grazing intensity exceeded the threshold, and the adaptability of *A. inebrians* to grazing environment was stronger than that of *Stipa purpurea* (Zhang et al., 2022). However, moderate trampling of livestock can stimulate and enhance the tillering ability of Gramineae in CSF, the density and coverage are bound to increase, and the ecological conditions in the grassland also change, and the growth of *A. inebrians* is inhibited with the weakening of light, and it will even be expelled from the grass for a long time. In addition, there are few studies on field seed yield in *A. inebrians*, so it cannot be compared with other literature.

As an important part of community structure, grassland plant community diversity plays a vital role in maintaining grassland ecosystem stability and productivity (Song et al., 2018). In this study, the Pielou index was higher for CSF and the Simpson index higher for FOP, indicating that the grassland community composition included fewer weeds, excellent forage grew well and was evenly distributed for CSF, while more weeds invaded FOP. This is likely because Gramineae and Leguminosae with good palatability were eaten first in FOP, which provided a better microenvironment and growth opportunities for some small non-dominant plant species, especially weeds. In addition, frequent grazing provided greater opportunities for livestock hair and hooves to carry and transfer external plant propagules (e.g., seeds and reproductive organs). Studies have also shown that continuous grazing leads to soil compactness, nutrient loss, inhibits compensatory growth of edible pasture, increases the proportion of inferior pasture, and decreases the diversity of community species, while reasonable grazing weakens the growth of dominant species in the community, provides survival opportunities for inferior species, increases community diversity, and then increases system productivity, which is not completely consistent with the our results (Zhang et al., 2022). Therefore, ecosystem function is closely linked to its biodiversity, but biodiversity does not necessarily provide the grazing services provided by ecosystems (Msadek, 2021).

### 4.2 Effects of grazing system on soil physicochemical properties and enzyme activity

Grassland soil is an important basic factor in the grassland ecosystem, and grassland vegetation change is closely related to changes in grassland soil fertility and activity, which affect each other (Merbold et al., 2014). Our study showed that soil pH increased with deeper soil, because plant roots were mostly concentrated in the 0–20 cm soil layer, so microorganisms were enriched in rhizosphere soil, and the respiration of root and rhizosphere microorganisms produces CO_2_ that, combined with rhizosphere water, resulted in lower pH in the surface soil. The soil water content was enhanced and soil bulk density was reduced in CSF, because CSF was rested from grazing in the warm season; grass resources were abundant when grazing in October, grassland vegetation coverage was enhanced and livestock feeding time was shortened. When coupled with the low temperature in the cold season, bedding time was prolonged and walking time was shortened, which reduced the damage of livestock hooves to the turf and the soil water dispersion loss (Wang, 2017).

In terms of soil nutrients, we found that the content of soil total and available nutrients was low in FOP, because the amount of livestock gnawing and trampling rose with the increased grazing intensity, which decreases grassland primary productivity, existing stock of grassland plants and litter decomposition, thereby reducing soil nutrient content (Li, 2022). The non-growing season supplementary feeding and bedding scatter manure layer about 2 cm on the surface in CSF, and when the temperature increases in spring and the snow melts, it has favorable conditions, and the manure layer can decompose to produce a large amount of humus, which is conducive to the formation of soil aggregate structure and microbial activity, thereby improving soil physicochemical properties and fertility. As we all know, sheep’s urine is rich in nitrogen fertilizer, which creates favorable nutritional conditions for the growth and development of Gramineae with high feed value, this is consistent with the view that grazing livestock trampling, and return of manure and urine had positive effects on soil nutrient improvement (Wang et al., 2008). So the response of soil physicochemical factors to grazing is uncertain. In addition, Sun et al. (2016) found that grassland degradation presented synchronous degradation of aboveground communities and soil, consistent with our conclusions.

Soil enzyme activity is related to microbial metabolic processes and biochemical cycles of nutrients. In this study, the alkaline phosphatase, urease, and sucrase activities were high in CSF, consistent with the results of Qin et al. (2014) showing that increasing grassland degradation in the Qilian Mountains could gradually reduce soil enzyme activities. The reasons are that soil enzymes are mainly derived from soil microorganisms, plant root exudates and animal and plant residues, continuous grazing in four seasons intensifies grassland degradation, affects plant root aggregation and lowers the amount and activity of soil enzymes (Zhang et al., 2015). Especially entering the grass withering period, the bedding time was shortened, the feeding and walking time was prolonged to eat food-loving plants, and there was even damaged or exposured of the hypocotyl or plumule of seedlings in the cold season. However, urease and sucrose activities in CSF were significantly higher than in FOP, because the high aboveground biomass increased the plant roots in the 0–10 cm soil layer, coupled with less soil compaction and good permeability, and so enhanced soil nutrient utilization, microbial activity and reproductive capacity (Poeplau, 2016). The catalase activity is involved in conversion of soil matter and energy, its activity can characterize the strength of soil biological oxidation process and it showed no significant change in CSF and FOP.

### 4.3 Effects of grazing system on soil microbial community structure

Soil microbes play an important role in grassland ecosystems. Grazing affects changes in the composition of soil microbial communities that may have direct or lasting effects on ecosystems (Jing et al., 2022). The bacteria with the highest relative abundance in CSF and FOP is Proteobacteria at the phyla level, showing CSF higher than FOP. Since Proteobacteria is the most common phylum in the world, and includes important soil bacteria, with its metabolic activity the most important bacterial activity in soil (Iino et al., 2010), which plays an important role in soil improvement, can degrade waste in the soil, not only can increase the soil nitrogen content, but also reflect the soil quality, so CSF treatment can improve the soil quality of of poisonous weed-type degraded grassland (Lin, 2017). The dominant genera in CSF and FOP were Sphingomonas and 67_14 for bacteria, and Mortierella for fungi. However, their relative abundances varied in the different pastures, which was partly consistent with the results from the degraded grasslands of the Qinghai-Tibet Plateau (Wang et al., 2022). This indicated that although the effects of seasonal grazing on soil microbial community composition differed, dominant microbiota were the same. Basidiomycota and Ascomycota predominate in environments with high soil lignin content and are some of the main decomposers of lignified vegetation debris (Araujo et al., 2017), which may be one important reason for Basidiomycota and Ascomycota becoming the dominant fungal phyla. Finally, there were characteristic microorganisms for CSF and FOP, indicating that grazing in different seasons had different effects on soil microbial species.

Soil microbial diversity is a key indicator reflecting the soil’s ecological characteristics. We found that the bacterial α-diversity was higher than that of fungi in CSF and FOP, indicating bacterial dominance in the soil, which may be due to the trampling of livestock made the soil more compact, the aeration decreased, the number of gas-repellent bacteria increased slightly, and the bacteria’s ability to adapt to poor environments led to the occurrence of this phenomenon (Yin et al., 2020; Wang et al., 2022). The α-diversity of bacteria and fungi differed only in shallow soil, likely due to the gnawing of livestock allocating more assimilated carbon to roots (Hou and Yang, 2006), while plant roots were mostly concentrated in shallow soil, which stimulates growth and activity of rhizosphere heterotrophic microorganisms. Besides, FOP increased grazing disturbance, had a strong spatial heterogeneity in the sample communities, and the sample information was more dispersed, resulting in greater differences in the abundance and diversity of soil microorganisms within the group, while soil samples had lower heterogeneity in CSF. The Principal coordinate analysis of β-diversity showed significant differences in fungal communities between the two pastures, possibly related to plant species composition differences, species composition variation of grassland vegetation may cause changes of litter and root exudate components, and soil fungal community structure is indirectly affected available substrate (Yin et al., 2020). Studies have also shown that differences in plant diversity mainly affect bacterial rather than fungal β-diversity (Prober et al., 2014).

### 4.4 Relationships among soil microbial community, vegetation traits and soil physicochemical characteristics under different grazing systems

Different grazing management of poisonous weed-type degraded grasslands cause successional changes in grassland vegetation community structure in alpine meadows, which then lead to soil characteristics changes, resulting in changes soil microbial community structure and diversity and finally feeding back to degraded grassland vegetation communities (Li, 2022), making the relationships among them more complex.

In this study, there were significant differences in plant community composition, soil physical properties, nutrients and organic matter. These factors were the main drivers of changes in the diversity and composition of soil bacterial and fungal flora, and there were differences in their correlations. Variations in plant species affect soil microbial communities by altering the number and diversity of soil rhizosphere exudates, the increase of aboveground biomass of grassland vegetation significantly affects the amount of litter input to the soil, increases the nutrient content of the surface soil, and is of great significance to its dynamic change (Zhang C. et al., 2019; Wang et al., 2022). And soil nutrient content can affect the population and distribution of soil microorganisms, soil microbial habitat change is directly related to changes in soil physical properties (Li, 2022). In addition, soil bacteria and soil organic matter had a high correlation, Milchunas and Lauenroth (1993) also found that grazing affects soil microbiota abundance by changing the amount of organic matter returned to soil. Soil fungi had a high positive correlation with the *A. inebrians* population, which may be related to the fact that *A. inebrians* itself is the host and mutualist symbiont of the *Epichloë* endophyte. Therefore, the grazing system plays an important role in changing the vegetation community’s composition and soil characteristics, and causing changes in the soil microbial community’s structure and composition (Turatsinze et al., 2021).

The effects of environmental factors on soil microorganisms are complex, and these factors also have complex interactions. When these factors are combined with other factors, such as precipitation, temperature and human disturbance, the interactions among them are more variable. From the perspective of vegetation–soil–microorganism interrelationships, the influence of different grazing systems on poisonous weed-type degraded grassland was studied, and appropriate grazing management strategies were proposed, which will be a vital part of future alpine grassland degradation research.

## 5 Conclusion

In the cold-season grazing plus supplementary feeding pasture, the aboveground biomass, coverage and reproduction of *Achnatherum inebrians* (Hance) Keng ex Tzvele were significantly inhibited, and the coverage and aboveground biomass of edible forage were significantly increased. And long-term utilization plus supplementary feeding in cold season had positive effects on grassland communities Simpson and Pielou indexes, soil water content, total nitrogen, total phosphorus and available nutrients. Moderate grazing in the cold season affected community structure and interspecific relationships, and dominant species were inhibited, providing opportunities for the growth and development of inferior species. The non-growing season supplementary feeding and bedding help soil agglomerate structure formation and microbial activity, improve soil traits, and increase soil fertility. Soil microbial communities were also affected by grazing systems, with a higher abundance of bacterial dominant species but the lower abundance of fungal dominant species in the cold-season plus supplementary feeding pasture. And only soil fungal community diversity differed significantly between the two pastures. Different grazing systems have a greater impact on vegetation and soil characteristics than microorganisms, and the response of soil microorganisms to grazing systems may be time-lagging and inconsistent, and bacterial and fungal communities respond differently. Therefore, grazing and supplementary feeding in the cold season treatment can effectively inhibit the growth of *A. inebrians*, restore edible pasture, and improve soil nutrients, which is particularly important for the *A. inebrians*-type degraded grassland ecosystem.

## Data availability statement

The datasets presented in this study can be found in online repositories. The names of the repository/repositories and accession number(s) can be found below: BioProject, PRJNA1013135, https://www.ncbi.nlm.nih.gov/bioproject/?term=PRJNA1013135.

## Author contributions

YC: investigation, data curation, methodology, writing – original draft, writing – review & editing. CX: investigation. KM: investigation. QH: supervision, writing – review & editing. XY: supervision, funding acquisition, writing – review & editing.
